# Management and prognosis of pancreatic cancer over a 30-year period

**DOI:** 10.1038/sj.bjc.6605150

**Published:** 2009-06-30

**Authors:** M David, C Lepage, J-L Jouve, V Jooste, M Chauvenet, J Faivre, A-M Bouvier

**Affiliations:** 1 Inserm U866, Registre des cancers digestifs, Dijon, BP 87900 F-21079, France; Université de Bourgogne, Dijon, F-21079; CHU, F-21079, Dijon cedex, France

**Keywords:** pancreatic cancer, cancer registry, management, stage at diagnosis, survival

## Abstract

BACKGROUND: The aim of this study was to report on changes in the diagnostic assessment, patterns of care and survival over time for pancreatic cancers.

METHODS: A total of 2986 cases of pancreatic cancer from the Digestive Cancer Registry of Burgundy (France) over a 30-year period (1976–2005) were considered. Non-conditional logistic regressions were carried out to identify the factors associated with resection for cure and with the use of chemotherapy. A multivariate relative survival analysis was carried out.

RESULTS: Diagnostic procedures have changed. Ultrasonography and computed tomography progressively have become the major diagnostic procedures. There was a slight improvement in stage: the proportion of stage I–II was 2.8% in the 1976–1980 period and 8.8% in the 2001–2005 period (*P*<0.001). There was a similar trend in the proportion of cases resected for cure, the corresponding percentages being 4.5 and 11.3%, respectively (*P*<0.001). The 5-year relative survival increased from 2.0 to 4.2% (*P*<0.001). In the multivariate relative survival analysis, the period remained a significant prognostic factor. Stage, sex, age and histology were independent prognostic factors.

CONCLUSION: Over a 30-year period, there were minor changes in the stage at diagnosis, resection for cure and prognosis of pancreatic cancers, although there were improvements in the diagnostic modalities. Pancreatic cancer still represents a major challenge in oncology.

Pancreatic cancer is currently generating particular interest because of its increasing incidence and its particularly poor prognosis. Numerous developments have taken place in post-operative resuscitation and non-surgical treatments. These were initiated in specialised centres and have progressively spread. Population-based studies that include all of the cases arising in a well-defined population are the best way to assess the real management of these cancers. The objective of this study was to draw a picture of the trends in pre-therapeutic evaluation, stage at diagnosis, treatment and survival of pancreatic cancers in a well-defined French population over the past 30 years.

## Materials and methods

The population-based digestive cancer registry of Burgundy (France) records all digestive tract cancers diagnosed in the resident population of two administrative areas (1 050 000 according to the 1999 census). Cancer extension at the time of diagnosis was classified according to the TNM classification. Three stages were defined: stage I–II (T1–4 N0 M0), stage III (N1 M0) and stage IV (M1). Non-resected cancers with no evidence of visceral metastasis were grouped into stage IV and classified as advanced cases (*n*=1301). Those who underwent resection, but were not staged, were classified as unknown (*n*=9). The life status was known for 2968 patients (99.0%) in January 2008.

Relative survival was calculated. The excess hazard ratio of death was estimated using a relative survival model based on the general linear model. A total of 2986 pancreatic cancers were considered. Ampullary, intrapancreatic bile duct and duodenal cancers were excluded.

## Results

### Diagnostic modalities

**Figure 1:**
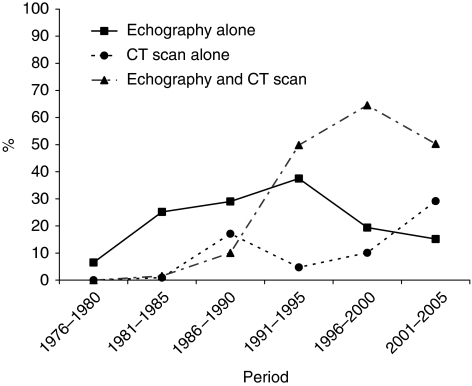
Evolution of imaging in diagnosis strategy

Diagnostic procedures varied over time (Table 1, Figure 1). There was a dramatic decrease in the proportion of cases diagnosed at the time of laparotomy. The diagnosis was based on surgical findings in 37.4% of the cases over the 1976–1980 period, and in 3.5% of the cases over the 2001–2005 period. Laparoscopy as a diagnostic tool was used until 1985: 31.3% of patients between 1976 and 1980, and in 13.9% of cases between 1981 and 1985. The proportion of patients who had an abdominal ultrasound (US) examination increased from 6.5% (1976–1980) to 87.3% (1991–1995), then tended to decrease. Computed tomography (CT) scans were introduced in 1981. It was carried out in 2.4% of the cases over the 1981–1985 period and in 79.5% over the 2001–2005 period (*P*<0.001). Endoscopic ultrasonography emerged in the early 90s and was carried out in 28.5% of cases during the 2001–2005 period. The use of MRI, which was available since 1991, remained infrequent.

### Stage at diagnosis and treatment

The proportion of stages I and II increased over the first four study periods from 2.8 to 10.4% (*P*<0.001), then levelled out. Most cases of pancreatic cancers were advanced cases.

The proportion of patients who underwent resection for cure was stable over the first two study periods, increased between the third (1986–1990) and the fourth (1991–1995), and then remained stable (*P*<0.001) (Table 1). The proportion of these patients receiving adjuvant chemotherapy increased from 5.0% (1986–1990) to 40.2% (2001–2005). Overall, 22.7% of the patients aged <65 years were resected for cure. This decreased to 13.7% of those aged from 65 to 74 years and to 7.8% of patients aged >75 years (*P*<0.001). Operative mortality after surgery for cure decreased significantly over the study period. It was 28.0% over the 1976–1985 period, 9.5% over the 1986–1995 period and 5.1% over the 1996–2005 period (*P*<0.001).

Chemotherapy was carried out after resection for cure in 19.4% of the cases. This proportion was 41.8% during the last study period. Chemotherapy was carried out in 30.4% of the cases after palliative resection and in 16.6% in non-resected cases (*P*<0.001). Before 1990, palliative chemotherapy was hardly used (3.5% of cases). The proportion of patients receiving palliative chemotherapy increased from 10.4% (1991–1995) to 41.8% in the last study period (*P*<0.001). Radiotherapy was also used as a palliative treatment. It emerged after the first period and remained rarely used ever since.

### Survival

Overall 1- and 5-year relative survival rates were, respectively, 19.8 and 4.1%. Survival was higher in females than in males (*P*<0.001), in patients under 65 years than in older patients (*P*<0.001) and in endocrine tumours compared with the other histological types (*P*<0.001) (Table 2). The 5-year relative survival increased from 2.0 to 4.2% between the first and the last time periods (*P*<0.001). Stage was the most important determinant of survival. The 5-year relative survival rate was 25.7% after resection for cure and 2.0% for the other treatment modalities (*P*<0.001).

In the multivariate relative survival analysis, sex, age, histological type, stage and period of diagnosis were significant prognostic factors.

## Discussion

The data presented here report the management and survival of pancreatic cancers at a population level over 30 years.

One point of interest of this study was to describe changes in the diagnostic assessment of pancreatic cancer. No comparable data are available in the literature. Until the early 1980s, exploration of the pancreas was difficult, which explains why diagnosis was often based on operative findings. This was the case in nearly 40% of cancers over the 1976–1985 period in our study. Diagnosis became easier with the development of abdominal US examination and then of CT. Both of these investigations were often carried out, in particular over the 1996–2000 period. In the most recent period, there was a trend to carry out CT directly. MRI imaging is still rarely used, whereas the importance of endoscopic ultrasonography is increasing. Thus, it is clear that the development of medical imaging has made diagnosis easier.

Changes in diagnostic strategies were not associated with a major improvement in the stage at diagnosis. The lateness of symptoms can explain why only limited improvements in the management of pancreatic cancer can be expected from the development of diagnostic strategies. The proportion of patients resected for cure increased from 4.5 to 11.3% and the proportion of those in stage I–II increased from 2.8 to 8.8%. The fact that improvements were seen in the first three 5-year periods, before levelling off during the three following periods, is disappointing. Resection rates for cure and stage were lower than those reported in hospital-based series (Bilimoria *et al*., 2007). Hospital data were provided by specialised units and as such cannot be used as a reference.

New approaches to the treatment of pancreatic cancer need to be found. The benefits of adjuvant treatment using chemotherapy and/or radiotherapy have been reported (Stocken *et al*., 2005). However, the benefits on survival, although significant, are modest. Palliative chemotherapy regimens also improve survival (Sultana *et al*., 2008). One of the most striking trends in the management of pancreatic cancer lies in the reduction of operative mortality, in particular after surgery for cure. It was 5% over the last 10-year period, close to figures reported by specialised teams. Major advances have been made by the way of the thorough evaluation of associated medical conditions and by improvements in post-operative resuscitation.

The prognosis of pancreatic cancer remained markedly grim, with <5% of survivors 5 years after diagnosis. The survival rates reported here are comparable with those of the US Surveillance Epidemiology and End Results database (Ries *et al*.), the EUROCARE database (Sant *et al*., 2003), and with the England and Wales national registry (Mitry *et al*., 2008). Stage was the major determinant of survival. Our data also showed a worse prognosis in men than in women. This had already been reported (Eloubeidi *et al*., 2006). At the same age, women probably have lesser co-morbidity than men. The much better prognosis of endocrine tumours has already been reported (Fesinmeyer *et al*., 2005). The development of these tumours is thought to be slow (Lepage *et al*., 2004). In this situation, aggressive treatments are justified.

As symptoms of pancreatic cancer appear late, it is necessary to make advances in a better understanding of the genetic and environmental causes, and of the mechanisms involved in its development. Smoking is the most consistently identified risk factor, although its role is not as important as for other cancers. The risk of pancreatic cancer seems to be greater in people having high-energy diets and lower in those having diets that are rich in fruits and vegetables. Detection of asymptomatic pancreatic cancers remains a challenge, as high-risk groups for pancreatic cancer are not sufficiently known. The association between diabetes and pancreatic cancer has long been recognised. However, the prevalence of pancreatic cancer is low. A reliable serological biomarker with a high likelihood of underlying asymptomatic pancreatic cancer is needed to yield a successful screening strategy. In addition, improvements in treatment are still needed.

## Figures and Tables

**Table 1:** Trends in diagnostic procedures, stage and management of pancreatic cancers (%)

	**1976–1980 (*N*=246)**	**1981–1985 (*N*=330)**	**1986–1990 (*N*=479)**	**1991–1995 (*N*=528)**	**1996–2000 (*N*=665)**	**2001–2005 (*N*=738)**
*Procedures used for diagnosis*						
Surgical findings	37.4	40.6	26.7	5.5	3.5	3.5
Cholangiography^a^	10.0	5.1	10.4	8.8	6.7	3.2
Ultrasonography	6.5	26.7	39.0	87.3	83.9	65.6
Computed tomography	0.0	2.4	27.1	54.6	74.6	79.7
Magnetic resonance imaging	—	—	—	1.1	10.5	9.1
Endoscopic ultrasonography	—	—	—	8.9	11.1	28.5
						
*Stage at diagnosis* b						
I/II	2.8	4.6	7.8	10.4	8.0	8.8
III	2.0	1.2	2.5	4.8	6.3	5.7
Advanced^c^	95.2	94.2	89.7	84.8	85.7	85.5
						
*Treatment*						
Resection for cure	4.5	4.5	8.7	12.5	11.0	11.3
Palliative surgery	57.2	65.9	55.7	48.5	53.3	48.8
Palliative chemotherapy	1.7	3.5	4.4	10.4	28.5	41.8
Palliative radiotherapy	0.0	7.7	4.2	4.8	12.5	8.8
Best supportive care^d^	37.4	25.7	34.0	37.4	33.2	38.7

a Retrograde or trans-hepatic diagnostic cholangiography.

b Unknown: nine cases.

c Metastatic and/or non-resected cases.

d Including medical biliary stent.

**Table 2:** Prognostic factors for pancreatic cancers: univariate and multivariate relative survival analysis

		**Univariate analysis**		**Multivariate analysis**		
	***N***	**1 year**	**5 years**	**Hazard ratio**	**95% CI**	***P*-value**
Global	2968	19.8%	4.1%			
*Sex*						
Males	1652	18.3%	3.4%	1		
Females	1316	21.7%	5.1%	0.82	(0.76–0.89)	<0.001
						
*Age at diagnosis (years)*						
<65	809	29.4%	7.6%	1		
65–74	922	19.8%	3.5%	1.22	(1.10–1.35)	<0.001
75–84	901	15.5%	2.7%	1.33	(1.20–1.48)	<0.001
⩾85	336	7.5%	0.0%	1.98	(1.71–2.30)	<0.001
*Period of diagnosis*						
1976–1980	246	9.5%	2.0%	1		
1981–1985	324	15.8%	4.5%	0.67	(0.56–0.79)	<0.001
1986–1990	471	18.6%	3.7%	0.68	(0.58–0.80)	<0.001
1991–1995	527	16.8%	4.7%	0.79	(0.67–0.92)	0.003
1996–2000	664	22.5%	4.4%	0.62	(0.53–0.72)	<0.001
2001–2005	736	25.6%	4.2%	0.61	(0.52–0.71)	<0.001
						
*Stage* a						
I-II	231	59.2 %	24.7 %	1		
III	129	50.0%	12.7 %	1.44	(1.12–1.84)	
Advanced^b^	2599	14.9 %	2.0 %	3.04	(2.56–3.60)	<0.001
*Morphology* c						
No histology	1382	14.2%	1.8%	1		
Adenocarcinoma	1375	23.1%	3.7%	0.24	(0.16–0.34)	<0.001
Malignant endocrine tumour	82	64.4%	47.1%	1.08	(0.88–1.34)	<0.463
Others^c^	129	16.8%	6.7%	0.97	(0.89–1.07)	0.550

a Metastatic and/or non-resected cases.

b Unknown: nine cases.

c Epidermoid cancer: 8 cases, lymphoma: 10 cases, undifferentiated carcinoma: 70 cases, sarcoma: 5 cases, cystadenocarcinoma: 36 cases.
